# Impossible futures and the ethics of hopelessness: learnings from Black liberation system entanglements part III

**DOI:** 10.3389/fsoc.2026.1762730

**Published:** 2026-06-22

**Authors:** Victor Udoewa

**Affiliations:** 1 International Computer Science Institute, Berkeley, CA, United States; 2 Bloom Works, Washington, DC, United States

**Keywords:** black liberation, decolonial futures, epistemic positionality, ethics of hopelessness, impossible futures, methodological reflexivity, social justice

## Abstract

Black Liberation work focuses on the flourishing of people of African descent across interconnected life components—community, health, education, employment, nutrition, and housing. Because these components are porous, connected systems, Black Liberation and social justice work are projects of system Futures. Yet what justice workers can or cannot imagine is deeply entangled with our experience as parts of these wounded systems. The resilience of systems of injustice has produced a future orientation of Afropessimism and Afronihilism within the African diaspora. White or Eurocentric Futures and design employ an ethics of hope that does not work for communities without hope, making these dominant paradigms exclusive. Futures has always been practiced by communities since the beginning of human and ecological life, especially by oppressed and decolonial movements. Viewing Futures as a spiritual practice, this paper uses a Black Liberation theology lens, informed by intersectional liberation theologies, to critique White Futures practice and its ethics of hope. This paper then presents how Black and underutilized communities that include people without hope practice futurism through an ethics of hopelessness founded in Impossible Futures. Rather than using impossibility as a euphemism or temporary state, Black Liberation and social justice workers mean it literally—a future that will never happen. This practice is called Black Liberation Futures Design, one contextualized glimpse of pluriversal Futures practices. This paper also makes a methodological argument for sociological research on race. Dominant Futures methodologies embed epistemological assumptions—about hope, agency, and linear temporality—that function as hidden scholastic bias, reproducing the racial hierarchies they claim to study or disrupt. Drawing on standpoint theory, Black Liberation theology, and lived experience, this paper shows how researcher positionality and the categories embedded in research practice shape what knowledge about race and racialization becomes possible. Black Liberation Futures Design offers sociology a reflexive, community-rooted methodological alternative for studying race, temporality, and structural oppression.

## Introduction

I am an interworld walker occupying multiple spaces and worlds, which often coexist inside of me. I am a Christian, male, heterosexual, U.S. designer person. Simultaneously, I am a U.S. Black American, invisibly disabled, immigrant-family, Nigerian person from a minority African Indigenous people group on both sides of my family, the Ibibio. My Ibibio name, *Anietie*, is a shortened version of the contemplation and question Who Is Like God? In the world of Ibibio people, time, past, present, and future function differently. In fact, we do not even have a single word for time, and we would need to hear the use of time in its full context in order to translate it to our language. This paper explores some of our experience with understanding of time. The word ‘I’ refers to the author. Depending on the context, I use the words ‘we’ or ‘our’ to refer to the readers and me, Ibibio peoples, African diasporic peoples, or community groups of whom I am a part and with whom I work. I specify the use of ‘we’ in each paragraph. When I speak about Black Liberation, I do so from the experiences of U.S. African-Americans, Nigerians, and other African diasporic people in places I have lived with various community groups (South Africa, U.K., etc.).

When a community group or community-based organization hires a social impact designer or futurist, the hired professional helps envision and design a new future for the community group. Very often, the designer or futurist creates a vision that the community does not own, like, or want and cannot use nor advance (Sheraz, 2021; Udoewa, 2022). This purchased future is a type of borrowed, used, or colonized future (Stein et al., 2020; Inayatullah, 2008, 2006). A borrowed, used, or colonized future is a future that has been imposed from the outside or uncritically adopted from dominant powers such that the future does not reflect the internal values, aspirations, or dreams of the community. One reason designers and futurists create borrowed futures is that Eurocentric Futures methods often do not fit or work for underutilized communities, thereby colonizing community futures.^1^

One way that futurists and social impact designers design and imagine borrowed futures for community groups is by creating futures based on hope and possibility. There are definitely members of community groups, social justice workers, and those working for Black Liberation who have hope. There are also members of those same groups who do not have hope. Those without hope may regard a liberatory future as an impossibility. How then does a heterogeneous community, including individuals without hope who see no possibility, use Futures activities, methods, and methodologies fundamentally based on hope, agency, and possibility?

Even when futurists and scholars talk of impossible futures, they do not mean impossible. Impossibility may be just a framing, what is unforeseen or unpredicted, creating fantastical future stories, theoretical mathematical frameworks focused on the limitations of computational behaviors for certain types of systems, apocalyptic eco-futures, the possible beyond our understanding of what is possible, the space between the possible and impossible, implausibility, infeasibility, or a necessary component of radical hope (Damhof and Gulmans, 2023; Goodale, 2023; Vandevoordt and Fleischmann, 2021; Bradbury, 2017; Hurley, 2017; Chen et al., 2015; Gough, 2010; Fischer-Kowalski et al., 2007; Higgins, 2007). In contrast, in my communities and in this paper, impossibility is not a framing or a temporary state until people can see clearly; impossible literally means that the future will never happen. For many justice workers who are without hope, the just future they desire can and will never ever happen.

What then is a community group supposed to do when engaging with Futures methodologies? Are they only left with exclusive Eurocentric Futures practices that use a prerequisite of hope and possibility, ostracizing or ignoring those who see no possibility and have no hope? Is there any type of ethic inclusive of people with hope and people without hope so that all may participate in participatory Futures work? Is there any reason or way to move forward in action toward a future that will never be? These are the questions I explore from my African and African-American social location and ontologies, worlds, or realities.

This paper also addresses a methodological problem that sits at the heart of sociological research on race and racism. The theoretical and methodological tools sociologists use to study race are never neutral; they carry embedded epistemological assumptions that reproduce categories, hierarchies, and worldviews. Scholars have drawn attention to the need for epistemic reflexivity in race research—an awareness of the scholastic bias introduced by the categories, techniques, and theories researchers bring to their work (Ba, 2025; Kim, 2025). This paper demonstrates that dominant Futures methodologies carry precisely such a bias: they embed hope, agency, and linear temporality as prerequisites for participation, thereby rendering invisible the structural conditions that produce Afropessimism and Afronihilism (Cascelli and Blanke, 2026; Ferguson et al., 2026; Tekogul, 2023). By drawing on sociology’s core analytical focus on social structures, processes, and meanings, and integrating insights from Black Liberation theology, Futures studies, and Afropessimist scholarship, this paper shows how the ethics of hopelessness and Black Liberation Futures Design offer researchers a reflexive methodological orientation for working with racialized communities whose relationship to the future is shaped by centuries of structural foreclosure. The positionality of the researcher—and the epistemological assumptions baked into their methods—determines whose temporal experience becomes legible and whose is rendered a barrier to participation.

This exploration is part of a series of papers on a systems practice in which we engage in Black Liberation work. In part I of this paper series, I lightly described the beginning of a healing-centered, decolonial approach to system health that uses a hospice and end-of-life doula sensibility for systems practice linked to a midwifery and birthing doula approach to system health (Udoewa, 2023). Part II built on Eurocentric, traditional knowledge of system traps and shared an updated understanding of system traps, archetypes, and entanglements justice workers experience in Black Liberation and social justice work (Udoewa, 2024a; Meadows, 2008; Braun, 2002; Kim, 1993). As justice workers, we question ourselves: if systems of injustice are so complex, adaptive, self-reorganizing, and resilient, despite our best efforts, how then do these persistent system traps, entanglements, and trauma responses affect our orientation toward the future? This is the question I share and explore in this paper, part III of the series.

I start the paper by sharing examples of Futures practice before the historicized start of Futures, decolonizing the history of Futures. Then, viewing Futures as a spiritual practice, I explore the components of Black Liberation Theology. Using Black Liberation Theology as a lens, we—the readers and I—critique Eurocentric or White Futures and show how it is built on an ethics of hope, creating an exclusive entryway point.^2^ We then explore Afropessimism and Afronihilism, illuminating how and why there are people without hope, for whom hope-based methodologies may not work. Lastly, we share a Black Liberation Futures practice, an inclusive practice that invites those without hope. It is a practice that has a different orientation to time, a different conception of Futures than the Futures cone, and a different path forward for movement toward a future that will never happen, especially for those without hope.

## Glimpses from an alternative history of futures

If you ask when did Futures begin, certain scholars might say it began in 1970 with Alvin and Heidi Toffler’s book Future Shock that popularized the wave metaphor for social and economic changes in the United States, similar to the Three Horizons framework (Toffler, 1984; Curry and Hodgson, 2008). Future Shock generated mainstream excitement and energy in future studies focused on the post-industrial economy. Other scholars might say Futures began in 1961 with Fred Polak’s book The Image of the Future that popularized imagining alternative futures (Polak, 1973). Still, others might cite (Jungk, 1954) book Tomorrow Is Already Here that critiqued the U.S.’s alleged colonization of the future and popularized the idea that the future is already here though not evenly distributed (Jungk, 1954). Others might point to post-World War II planning in the U.S., statistical forecasting in the 1920s, or futurism movements in Italy, the Soviet Union, or Japan (Gidley, 2017). There are even scholars that might point to Wells, 1914 book The War That Will End Wars, which certain scholars say established science fiction (Wells, 1914). Wells also used the English term ‘foresight’ in an address in 1932 (Wells, 1914).

However, colonialism is captivated by chronologies, the Eurocentric invention of a concept, and linearly developmental progress (Smith, 2022). What happens if we—the readers and I—define Futures, future studies, futurology, or futurism as simply the act of anticipating, forecasting, remembering, applying, exploring, analyzing, testing, evaluating, synthesizing, or creating the future or futures? With that understanding, we can see that communities have always practiced futuring. Let us decolonize the history of Futures and look at examples before the Eurocentrically historicized start of Futures work.

On August 14th of 1862, in one event in a broader, multicentury futures dialogue, U.S. President Abraham Lincoln invited five men, prominent in the Washington, DC African-American community, to a meeting about the future of African-Americans. John F. Cook, Jr., Edward Thomas, Benjamin McCoy, John T. Costin, and Cornelius Clark were all well-educated and well-affiliated African-American men in the D.C. antebellum elite (Masur, 2010). During the meeting Lincoln explained to the men that he believed physical difference between the races made separation preferable for both (Basler, 1953). Lincoln, a colonizationist or supporter of the emigration of African-Americans, tried to rally support from the men to send African-Americans to the Chiriquí region of what is now Panamá in Central America. Though this proposition was largely unpopular among African-Americans in DC and across the United States, it led to debates, especially among African-American communities in DC, around alternative futures. It also led to debates about Participatory Futures, specifically who in the African-American community is allowed to speak for others when evaluating and choosing different futures; this may be why the committee never issued a formal statement or decision (Masur, 2010). Ultimately, Lincoln’s request shows a lack of understanding of African-American indigeneity and that, for the majority of African-Americans, emigration was not their preferred future (Boateng, 2023; Brazelton, 2021; Mays, 2021; Gafney, 2017). Rather, they worked toward their preferred future—a multiracial society inside the United States.

We—the readers and I—can also travel to Africa where, for millions of years, families, groups, and small communities of people had always chosen a nomadic future of foraging. Though agriculture first developed in the Fertile Crescent of the Middle East in 9000 BC and later in China and New Guinea in 7000 BC, people in sub-Saharan Africa still did not adopt agriculture (Xiaoyun et al., 2012). In fact, in an act of pluriversal futures, foraging communities in southwestern Africa in the Kalahari Desert knew about farming as a possible, alternative future, but did not adopt this future for their world and reality; farming was not their preferred future (Baker, 2025). Evaluating various future scenarios, they knew farming required more hours of work, brought worse health, poorer nutrition, and more susceptibility to the weather and natural disasters. African fine-tuning meant that African humans had evolved and adapted to their environment over millions of years, able to successfully survive through foraging (Baker, 2025). Then around 3,000 BC, rejecting the binary of different futures, communities of West Africans chose a blended future—farming piecemeal as a way to feed domesticated animals they shepherded and bred as a food source (Bellwood, 2022). This opened up more possibilities, making full-time agriculture more feasible, and farming in West Africa grew from 3,000 to 1,000 BC starting with millet and sorghum and later rice (Ehret, 2002).

Futures practice reaches even further back, beyond the emergence of *Homo sapiens*. Early hominids engaged in a multigenerational futures project through stone tool development, iterating across deep time from Oldowan tools 2.6 million years ago through increasingly sophisticated blades and axes, each generation working toward a better-imagined future (Udoewa, 2022; Pruitt, 2019). This intergenerational futures work predates our species and reminds us that anticipating and building toward preferred futures is a feature of human and pre-human life. Nor is Futures practice limited to the human world: birds, whales, and other animals navigate thousands of miles based on seasonal predictions; squirrels and bees store food for future scarcity; underground fungal networks redistribute resources between plants based on long-term forest health needs (Wohlleben, 2016; Way and Montgomery, 2015; Woodall et al., 2009). Futures practices—anticipating, planning, and acting for futures yet to come—have been woven into ecological and social life long before any institution named and claimed them.

From the 3,100 BC unification of the Northern and Southern kingdoms of Egypt, oracle and divination systems in Africa, the Nyatsimba Mutota-led migration north to found the Mutapa empire, and the 17th century women warriors of Dahomey, to the 1803 Legend of the Flying Africans in the antebellum southern United States, the griots of West Africa, the U.S. American spirituals, and the various decolonial struggles for liberation across the diaspora, one thing is clear: Africans have always been futurists (Brooks, 2020). Therefore, humans have always been futurists.

These examples show intergenerational futures, blended futures, (non)participatory futures, preferred futures, pluriversal futures, alternative future evaluations, and much more across African history. These Futures practices evince the values of the various people groups, species, ecosystems, and landscapes who practice them.

## Black liberation theology

The presence of those values hints that Futures is a spiritual practice. Spirituality is the set of values, meanings, purposes, and understandings of one’s place in existence as well as the set of practices, rituals, and experiences that affect, challenge, alter, or enrich one’s existence, connections, purpose, interiority, meaning, and sets of values. Therefore, we can use a spiritual or values-laden lens when approaching Futures.

All design and Futures practice is already value-laden. However, if we, designers, do not explicitly choose those values, as in values-sensitive design, those values will still emerge implicitly (Friedman and Hendry, 2019). Because of my social location and because I am often doing Black Liberation work in communities that often have some Christian heritage or experience, we use Black Liberation Theology as our lens in approaching Futures. Before applying it to Futures, let us define Black Liberation Theology.

Black Liberation Theology, first described in the United States by James Cone, was one of a few different strains of Black Theology that developed in the U.S. in the late 1960s (Douglas, 2019). All types of Black Theology highlighted the political side of religion in historical and present-day Christianity and worked to reconcile the spiritual with the political, viewing White Theology as hypocritical (Norris, 2020; Douglas, 2019). While other types of Black Theology focused on Jesus being literally Black or heavily prioritized reconciliation, Cone’s Black Liberation Theology focused on ontological Blackness, or Blackness as a way of being (Cone, 2019). Black Liberation Theology is a marrying of the Black Power philosophy of Malcolm X with the public and political theology of Martin Luther King, Jr. (Cone, 2019, 1991).

Black Liberation Theology has several important components, a few of which I will mention. First, similar to Latin American Liberation Theology developed at the same time and its preferential option for the poor, God has a preferential option for the oppressed (Cone, 2025; Gutiérrez, 2023, 1970). God identifies with and works to liberate the oppressed, who in the U.S. at the time of Cone’s writing in 1970 and today, are Black people. Second, Jesus is a liberator working in solidarity with the oppressed and marginalized. Third, liberation from systemic oppression is God’s essential and salvific work. Fourth, therefore, God is Black. Though Blackness is an ontological symbol, it is literal in relation to human reality while symbolic in relation to the divine (Douglas, 2019, p. 62–66; Tillich, 2001). To be Christian theology, any U.S. American theology must contend with the Black experience (Cone, 2020). Fifth, to be free, you must become Black—rejecting the false comforts of neutrality, actively choosing the work of liberation, and adopting the theological and political commitments of those fighting systemic oppression.

Womanist theology and intersectional analyses have gone beyond, influenced, and improved Black Liberation Theology (Cannon, 2021; Williams, 2013; Grant, 1998). An intersectional womanist theology would say that God is not just Black. God is a Black woman (Cleveland, 2023; Johnson, 2023; Mahaffey, 2023; McIntosh, 2017; Polana, 2013). Even more, today in the U.S., such a theology might say God is a Black, transgender, female, Muslim, poor, immigrant—or anyone who stands in solidarity and works alongside Black, transgender, female, poor, Muslim, immigrants (Douglas, 2019).

Lastly, theology, in general, contains a Futures practice. The subset of theology dealing with the future is called eschatology—the theology of the future, or the study, belief, or understanding of God as it relates to the future. Black Liberation Theology uses a participatory eschatology. God does not create the future while African-Americans sit idly watching and waiting. The future shows the failings and ungodliness of the present, creating dissatisfaction with the present; therefore African-Americans and those who become Black participate in the creation of the future with God and all creation (Cone, 2019, p. 142).

## The failings of white design, white futures, and the ethics of Hope

Using a Black Liberation Theology lens, informed by other liberation theologies like womanist theology, lets us examine, Eurocentric design and Futures. The Human-centered Design (HCD) mindsets, Futures mindsets, and Futures principles provide material for us—the readers and I—to analyze (Faiella and Corazza, 2025; IDEO.org, 2025; Boldrini, 2022; Gorbis, 2019; McGonigal, 2022, 2019a, 2010; Hines et al., 2015; Cascio, 2008; Suddendorf and Corballis, 2007). Due to IDEO’s global influence and marketing of HCD, I mapped different Futures mindsets to the IDEO.org HCD mindsets and listed the Futures principles separately (Tables 1, 2).

**Table 1:** List of HCD and white Futures mindsets.

HCD mindsets	Futures mindsets
Learn from failure	
Make it	
Iterate	
Creative confidence	Creativity and instinct
Embrace ambiguity	Multiple futures
Empathy	(Hard) Empathy
Optimism	Urgent optimism, hope, mental flexibility, super-empowered hopeful individuals

**Table 2:** List of White Futures principles.

Futures principles
Multiple Futures, forget predictions
Look back to look forward
Uncover patterns
Focus on signals
Create a community

There are many problems with the Eurocentric design and Futures mindsets in the worlds that many people inhabit (Table 1). For instance, learning from failure, even in small ways, is a privilege that does not work for many communities. Failure can literally mean death due to violence, law enforcement, lack of access to health care, and more for many communities. The ‘make it’ mindset is a bit better because it does not prioritize the written word; however, for many hyperlocal and indigenous communities of which I am a part, it does not go far enough. For us, a more holistic mindset would include a variety of ways people know and learn—sense it, dance it, sing it, feel it, play it, embody it, sound it, emote it, and more. Similarly, the iterate mindset recognizes that it is hard to create the best design immediately, but it only focuses on time. For multiple people groups around the world, space and time are not separate concepts. We need to iterate across space as well.

The most important mindsets for our purposes are those that explicitly embody an ethics of hope (Table 1). First, according to these frameworks, designers and futurists should manifest creative confidence and listen to their instinct. Second, designers and futurists can gain empathy or hard empathy—a creative kind of empathy that helps a person know, understand, and imagine when that person has no first-hand experience of what people are experiencing (McGonigal, 2019a). In contrast, studies have shown men trail women in empathy (Mestre et al., 2009), the ability to have empathy decreases with income (Goleman, 2013; Lane, 2001), white people are least likely to show empathy (Newheiser and Olson, 2012; Nosek et al., 2002), and it is harder for a person to have empathy for other demographically dissimilar people (Mei et al., 2024; Newheiser and Olson, 2012; Nosek et al., 2002). Even though white, often male, designers and futurists are least likely to develop and maintain empathy, they are still confident they can develop and hold empathy and hard empathy (Ferai, 2024). Third, professional futurists and designers embrace ambiguity especially because the future is open and undecided. Fourth, professional designers and futurists are specifically optimistic and explicitly hopeful—urgently optimistic, full of hope, and mentally flexible. They are super-empowered, hopeful individuals (Cascio, 2008).

Professional Eurocentric, or White, designers and futurists use mindsets as a gateway. They teach the mindsets and facilitate practice of the mindsets because, according to their methodologies, participants need these mindsets in order to practice design and Futures (Staf, 2024). If a person does not have these mindsets, that person cannot practice design and Futures or their practice will be impeded, impaired, inadequate. These mindsets—urgent optimism, creative confidence, hard empathy, hope—are part of the ethics of hope. Therefore, without hope, a person cannot practice White Futures or White design.

What do White designers and futurists do if people do not carry this ethics of hope? White Futures has actual tactics, methods, and exercises to help participants reach the minimum level of optimism, hope, imagination, and mental flexibility. For instance, it is common for futurists to include a time horizon activity at the beginning of a Futures process. If participants struggle to imagine different futures from the present at a given time horizon, futurists just push the time horizon further out (Boulding, 1990, 2002; Johansen, 2012; Voros, 2003). Professional futurists also facilitate exercises to teach people how to imagine—exercises like hard empathy, predict the past, alternative histories, counterfactual thinking, and narrative forecasting (Sools, 2020; McGonigal, 2019a; Gioia et al., 2002). Professional futurists and scholars focus on developing the ability for mental simulation, mental flexibility, and mental time travel through activities like specificity training (Tanguay et al., 2023; Hallford et al., 2020; McGonigal, 2019b; Cuhls, 2017; Kaufman, 2010; Markley, 2008).

The confidence of White Futures often leads futurists to find fault with community members and workshop participants when the gateway mindsets are missing. In a recent gathering, professional futurists listed 14 different barriers preventing people from engaging with Futures work (McGonigal, 2016). It is fascinating to note that half of the barriers are problems with the people—their fear, their beliefs, their lack of belief in their agency, their lack of hope, their inability, their ideology, and their neurological issues (Table 3). The futurists at the gathering never considered that the problem might lie in their own Futures methodologies. This is a paradigmatic example of bias: the categories and techniques researchers and practitioners bring to their work quietly structure what counts as valid participation and whose experience becomes legible.

**Table 3:** List of barriers to Futures participation, identified by professional Futurists.

Seven barriers	Seven barriers
Lack of time due to urgent matters in the present	Lack of role models who think about the future
**Fear of being wrong**	Lack of exposure to futures thinking methods
**Belief that Futures thinking is irrelevant**	**Inability to see directions of change due to a lack of awareness of transformational forces**
Difficulty proving its value	**Rigid ideology**
**Lack of agency with respect to the future**	Pop culture overly influences the types of possible futures one imagines
**Lack of hope or optimism about the future**	Lack of time
Lack of encouragement to think imaginatively about the future	**Neurological hindrances**

**Figure 1:**
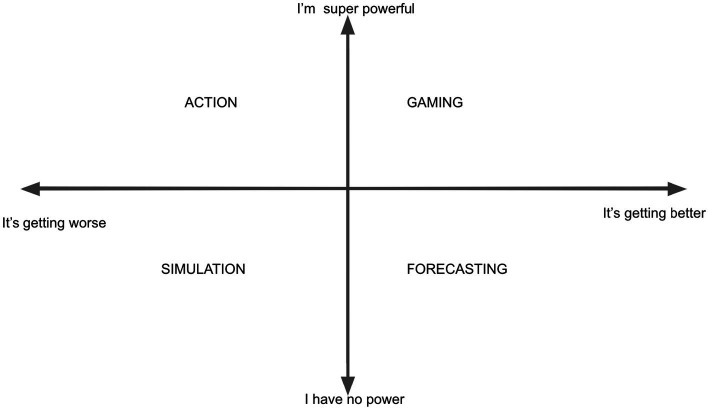
Futures four-square game and recommended set of White Futures methods.

In fact, many of the futurists at the gathering and other professional futurists use the Four Square game to determine what methodologies to use for each barrier (Hayward and Candy, 2020, 2017; McGonigal, 2016; Polak, 1973) (Figure 1). The goal is to create self-awareness in participants and, through further activities, help participants understand that they have agency and develop hope that the future can be better through their agency. If participants feel they have no agency and the future is getting worse, simulation methodologies can help participants experience potential, better, simulated futures and build their mental flexibility, imagination, and hope, moving them to the bottom right quadrant. If participants feel the future is getting better but they have no agency, forecasting methodologies showing open futures can illuminate the agency they actually have. If participants already feel powerful but feel the future is worsening, action-based methodologies incite them to use their power. Lastly, if participants already feel powerful and believe the future is improving, gaming methodologies can aid them in inviting others into their hopeful, optimistic, future-making work.

From a Black Liberation theological orientation there are two major problems with the White Futures approach. First, it is not a participatory approach like the participatory eschatology of Black Liberation Theology. Black Liberation Theology came from enslaved Christianity, the spirituals, the blues, and the practices and ways of being of Africans in slavery and after (Cone, 2025, 2022). Similarly, the practice of future-making in Black Liberation Theology is not created by God, but by the co-mingling and mixing of humans, animals, plants, ecosystems, and God together. White Futures, when interacting with communities with different ways of being and knowing, still often uses the same methodologies instead of allowing the Futures methodology to come from the ways of being, knowing, and Futures-doing the people already practice. Second, because professional futurists use the mindset and the ethics of hope as a gateway, it is an exclusive practice. James Cone said that in order to practice any Christianity in the context of the United States, you must become Black—an inclusive practice for Black people at the bottom of a hierarchy. One way of interpreting this call is that you must meet people where they are, working in solidarity with them, and taking on their slings, arrows, beatings, bruises, and goals as your own. Instead, White Futures violates this and exclusively asks people to become White, encoding White normativity as a baseline (Bell, 1995). If a person wants to practice White Futures, they must have hope, empathy, creative confidence, and optimism and be able to see multiple futures—become White. If they cannot, they cannot participate or their Futures work will be wounded and inadequate.

Do White futurists not realize there are people who, no matter how hard they try or practice, cannot see multiple futures (Table 2)? Do futurists understand that there are people for whom, when they look back, they cannot look forward? Do futurists see that there are people who only uncover patterns that point to repeating oppressive histories? Do futurists understand that they completely shut out and ostracize people who literally have no hope? These are not individual deficits or ‘barriers.’ They are structural effects—produced by the very systems of injustice that Futures work claims to address.

## Afropessimism, Afronihilism, and impossible futures

Various people groups have experienced hopelessness and impossibility across time and space. Long before any scholar named the experience in academic theory, people across the global African diaspora have experienced such hopelessness as a result of colonization, colonialism, neocolonialism, and the cyclical traps of injustice that make justice seem impossible (Udoewa, 2024a). In Black studies, scholars call this experience of structural impossibility, Afropessimism.

Afropessimism has a few core components. First, Blackness exists as a type of social death (Herrero and Wilderson, 2022; Wilderson, 2020; Patterson, 2018). Blackness is an ontological state of non-being, not just outside political life, but outside what it means to be human in Western colonial modernity. Because of this, anti-Black racism is not comparable to other forms of oppression, but is qualitatively distinct due to its non-humanness. It underpins and enables other forms of oppression like capitalism, colonialism, and more.

Second, anti-Blackness is foundational to society (Patterson, 2018). Anti-Black violence involves gratuitous or purposeless violence, natal alienation or severance from family and culture, and general dishonor or lack of recognition as a person. Black people experience this foundational violence not because of what they do, but due to who they are.

Third, within the existing systems, Black Liberation through rights, reform, progress, or inclusion, is structurally or systemically foreclosed (O'Donnell, 2020; Wilderson, 2018). Anti-Blackness is so foundational to the world that Black Liberation can only occur through an ontological break, apocalyptic rupture, complete transformation, or the end of the world (Fanon, 1970).

Afropessimism is a deep systems analysis based on the lived system experience of people at the bottom of a social hierarchy, a position that affords a knowledge advantage according to womanist standpoint theory (Suehn et al., 2023). Though Afropessimism has critics, it represents the understanding of certain, real Afro-descendant people (Hawkins, 2025; Olaloku-Teriba, 2018; Thomas, 2018). Because of the Afropessimist structural analysis and systems understanding, certain people even reach the emotional state of Afronihilism (Warren, 2018).

Rather than a system analysis, Afronihilism is an emotional and existential response to an Afropessimist analysis. Afronihilists reject hope (Warren, 2015). They have no hope or faith in progress or liberation and often critique weaker Afropessimistic analyses that leave space for possible, but improbable redemption. Afronihilists also embrace meaninglessness or nothingness, sometimes to the point of radical or existential despair (Brogdon, 2013). They accept that there is no absolute meaning or ultimate justice or any sense to this life and reality. They even reject any kind of construction of value or meaning under oppressive systems. Lastly, Afronihilists experience Black life as life in continual confrontation with death, disregard, invisibility, dehumanization, and negation.

Although there are weak forms of Afropessimism that allow for some action or even Afronihilists that find a hopeless creativity or artistry in the midst of despair, many people find no use in activist response, action, and progressive movement toward impossible futures (Terry, 2023; Havis, 2022, p. 74–75, 102; Ahmed, 2022; Ureña, 2021). How can White futurist practices and methods based on an ethic of hope work for Afronihilists experiencing radical despair and meaninglessness?

Imagine running Equitable Futures workshops with enslaved African-American women in 1686 using a 20 year time horizon. Imagine institutionalizing that workshop and repeating it every 20 years. Imagine, today in 2026, facilitating a White Futures workshop for African-American women in which you look back to look forward (Table 2). How could such an activity work? There are definitely African-American women who would be demotivated by the lack of structural progress. It is important to understand that the Afropessimistic critique is not saying that the system never changes. Afropessimism says that the position that Black people have in society has never changed and will never change. The same analysis is in scholar Derrick Bell’s racial realism theory which espouses the permanence of racism through peaks of progress (Bell, 1992a). Even though enslavement ended, it was followed by sharecropping, chain gangs, Black codes, vagrancy laws, and legalized apartheid in the U.S. American South, the era of terror—Jim and Jane Crow. Even though Jim and Jane Crow ended, it was followed by mass incarceration. The surface of the system changes, but the structure and outcomes remain the same in the lives of so many who experience a deep hopelessness (Udoewa, 2024a). They are unmotivated by such White Futures practices.

From a sociological methodological standpoint, this matters enormously. When sociologists and futurists design participatory research or community engagement processes around race, they routinely embed assumptions about participants’ capacity for hope, imagination, and future orientation. Eurocentric futurists and scholars fail to recognize hopelessness as a major orientation to the future, anthropologically or sociologically (Bryant and Knight, 2019). These assumptions, drawn from dominant Western methodological traditions, function as what Bourdieu and Wacquant (1992) call ‘the scholastic point of view’: the unexamined perspective of the researcher that gets imposed on the studied. Afropessimism and Afronihilism reveal that such assumptions are not neutral but racialized. Designing race research with communities whose temporal experience is shaped by structural foreclosure requires a different methodological starting point, one that does not pathologize hopelessness but recognizes it as a legitimate and structurally produced orientation toward the future.

## The ethics of hopelessness

Instead of an exclusive White Futures practice of becoming White, or making Futures participants go through the door of the tyranny of hope, futurists need to become Black. Futurists and designers need an ethic of hopelessness.

Latin American Liberation theologian and ethicist De La Torre (2017) calls hope a privilege that assuages the egos of those at the top of a social hierarchy. He calls for a theology of desperation that leads to hopelessness because when you are hopeless with nothing left to lose, you are forced to act. Unfortunately, that is not everyone’s experience. There are many people experiencing hopelessness in impossible futures who are not motivated to act or who are paralyzed in despair.

In contrast, when I talk about the philosophy, theology, or ethics of hopelessness, I mean theology and ethics that are unconnected to hope. I am talking about ethics founded on something much deeper than traditional hope, so that even people without hope are animated by it. Is this not what historians see in the U.S. Civil Rights movement of the 1960s? Civil rights activists did not say to themselves that when they protest by sitting at the segregated lunch counter, the servers will see the errors of their ways and change. They did not think that governors and legislators would see the value of their movement and act justly. No, it was the opposite. Through training, civil rights activists prepared for beatings, water hosing, dog attacks, and imprisonment. They were taught what to do when White people and authority figures said no, fought them, beat them, and abused them. They expected not to receive what they longed for—freedom.

I do not mean that they had no goal. They did have goals for Black and lower class liberation. In fact, the movement employed tacticians and strategists like Reverend James Lawson (Limbo, 2006). The activists, however, were not motivated by whether or not they could achieve the goal, whether the goal was possible. They had a slogan—we will not stop, until we succeed (Lakey, 2018). This means if we do not succeed, we will not stop; if we never succeed, we never stop. This slogan is in stark contrast to the burnout that certain contemporary social justice activists experience (Hanson, 2023; Chen and Gorski, 2015; Cox, 2011). Week after week, month after month, year after year, if such social justice activists see no change or movement toward the goal, they burn out. This happens because they are motivated by the goal itself. Once the goal is impossible, they lose energy, motivation, and hope.

An ethic of hopelessness functions differently. Because it is not based on the possibility of reaching the goal or hope, it is an inclusive ethic that invites the hopeful, the hope-unsure, the hope-ambivalent, and the hopeless—even those who believe the goal will never be achieved. With an ethic of hopelessness, we, Afro-descendant people, do not do what we do because we have hope. We do what we do because that is what integrity says we do. With an ethic of hopelessness, we do not do what we do because we believe we will see the outcome we want; we do what we do because it is the right thing to do. We do what we do because we want our children to look at us knowing we died doing the right thing. With an ethic of hopelessness, we do not do what we do because we believe we will achieve our goal; we do what we do because that is what dignity looks like. With an ethic of hopelessness, we do not do what we do because we have hope; we do what we do because that is what love does.

This sentiment fills the words of a great Afro-Caribbean prophet who proclaimed “We found love in a hopeless place” (Rihanna, 2011). If love is there, one might think it is not a hopeless place but hopeful. But it is the very conditions of hopelessness that allow love to show its integrity, steadfastness, and presence not conditioned by hope. This experience is even stronger in the words of one of the greatest African-American prophets (Houston, 1985).

*I decided long ago*

*Never to walk in anyone’s shadows.*

*If I fail, if I succeed.*

*At least I’ll live as I believe.*

*No matter what they take from me.*

*They cannot take away my dignity.*

Hope has nothing to do with the actions of the singer or songwriter. Dignity motivates her, not success. This type of freedom is not a goal, but a way of being. In other words, the ethics of hopelessness embodies an onto-liberationality—liberation as a way of being, not a destination, and not a direct fight against oppression. The ethic of hopelessness embodies a spirituals physicality, a blues sensibility, and a jazz spirituality. From the perspective of Western eyes, there are many spirituals that do not resolve lyrically—*sometimes I feel like a motherless child* … *a long way from home*. Likewise, in the blues, the lyrics never seem to resolve; the blues were a permanent state of being. Thus a blue sensibility is self-affirmation through song even when your situation is not changing and will never change. From the perspective of European classical music, the dissonance of jazz music also does not resolve (Miller, 2012). Speaking of African-Americans, jazz composer Duke Ellington said “Dissonance is our way of life. We are something apart, yet an integral part” (Matlin, 2022; Jones, 2015). In the world of African-Americans, the resolution of dissonance into consonance is not the goal: dissonance is a way of life. Similarly, liberation is not a destination or a goal, but a way of living with dignity in love in the in-between, the blue, the unresolved, the dissonant, without belief or hope in a different outcome.

The energy, motivation, and sustenance for liberatory work does not depend on the outcome. From a Black Liberation Theology perspective, liberation or liberatory work is not about winning or an analysis of the odds of winning (Cone, 2020, p. 81–82; Cone, 2019, p. 34). I can have zero success and still not experience burnout. This is because my energy and motivation do not come from outcome and results, but from living in alignment with my values, doing the right thing, embodying dignity, and doing what love does. Liberation is not a goal or a gift given to the oppressed; someone is free when they accept responsibility for their own acts (Cone, 2019, p. 32–33). With liberation as a way of being, though I may be beaten, bruised, jailed, or dying, I have the energy to move forward, in the mixed community of the hopeless, hope-unsure, and hopeful. For it is in community that values are chosen, because the community provides the structure in which our being as persons is realized (Cone, 2020, p. 103).

The ethics of hopelessness highlights the difference between faith and belief and their relationship to doubt. A person can fully doubt, fully lack belief in an impossible future, and still move forward in faith, putting their trust in a community that carries values of love, dignity, and justice, trusting that movement toward an impossibility is the best way to live, the right way to live. Faith can be present in the absence of belief. Faith builds upon doubt, through relational trust.

An ethic of hopelessness even paves the way to transformative, asset-based Futures. For example, non-disabled White futurists often imagine a world in which technology eliminates all disability because disability is not consistent with utopic futurity (Woodcock, 2009; Gleeson, 2005, 1998). With an ethic of hopelessness, we do not hope for a future in which disability ceases to exist. In community with able-bodied and disabled groups of color who experience the social construction of disability, we understand there is no future without disability. Disability is a part of life and inextricably linked to the experience of life as people move in and out of different abilities across their life. Additionally, disability is reified by its socio-political construction and social oppression, especially for Black, Indigenous, and people of color (Foreman-Murray, 2022; Frederick and Shifrer, 2019). Instead of hoping for a future without disability, dreaming of a Black disabled future is a radical act and a liberatory way of being (Drew and Wortham, 2021, p. 51).

Hopelessness is always proximate or related to some definition of hope. I have focused on Eurocentric hope—a positive anticipation and solid expectation of what one believes or for something in the future. However, there are many pluriversal understandings of hope. For instance, hope is a discipline, an ontological need, or the everyday practice of resurgence (Kaba, 2021; Chew et al., 2019; Freire, 2021). Hope can even be rooted in the past—in ancestral relationships and intergenerational continuity—or be a colonial tool, which resonates with Impossible Futures (Lindroth and Sinevaara-Niskanen, 2022; Grande, 2004). The ethics of hopelessness does not oppose these richer, practice-based, or ancestrally-grounded conceptions of hope; rather, it includes everyone, even those who have or lack these types of hopes.

Taking an asset-based approach, what possible redefinition of hope describes what Black Liberation and impossible futurists carry, instead of lack? It is based on the relationship between faith, hope, and love (Common English Bible, 2011, 1 Corinthians 13). Love is the dedication, prioritization, decision, and commitment to the world, to take action to be in right relationship with the world. Faith is the action taken, fueled by love, especially and specifically when you doubt and do not believe the future world you want will come to pass. Redefining hope, HOPE is the exhaustive, restful sleep you experience at the end of a hard day of faithful work fueled by love. It is not restful because you think or believe the future you want can or will happen. HOPE is restful because you have done good, hard, faithful work in alignment with intergenerational values. You rest easy knowing you have lived with dignity in love.

## Black liberation Futures design

### The irrelevance of the Futures Cone

The ethics of hopelessness in Impossible Futures creates a foundation for a Black Liberation Futures practice that disrupts colonial cartographies of time (Koselleck, 2004; Habermas, 1990). In the West, the growing consciousness, interest, and study of trauma has also participated in disrupting Western time, as trauma demonstrates how the past and future can be very present. However, a Western, clinical, psychopathological understanding views trauma as an inability to move beyond the past or being stuck in the past (Habermas et al., 2008). For African Indigenous groups, my Ibibio people, and even Black Liberation workers, being enmeshed and entangled in the past is neutral; the question is what kind of entanglement do you have to the past (Udoewa, 2023). For our progressive work is deeply conservative. We walk backwards to the future, reinvigorating precolonial convivialities.

**Figure 2:**
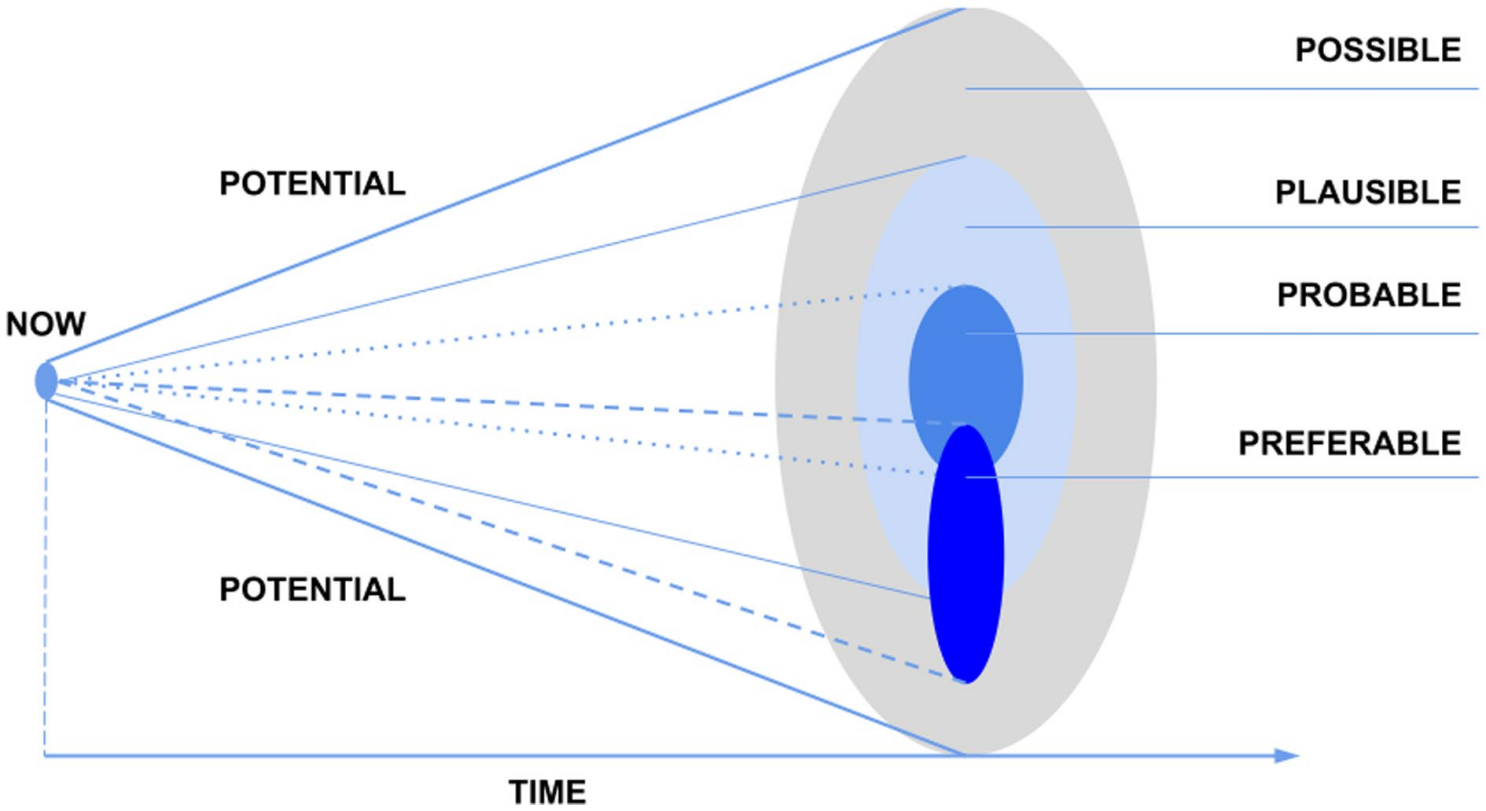
The Futures Cone.

**Figure 3:**
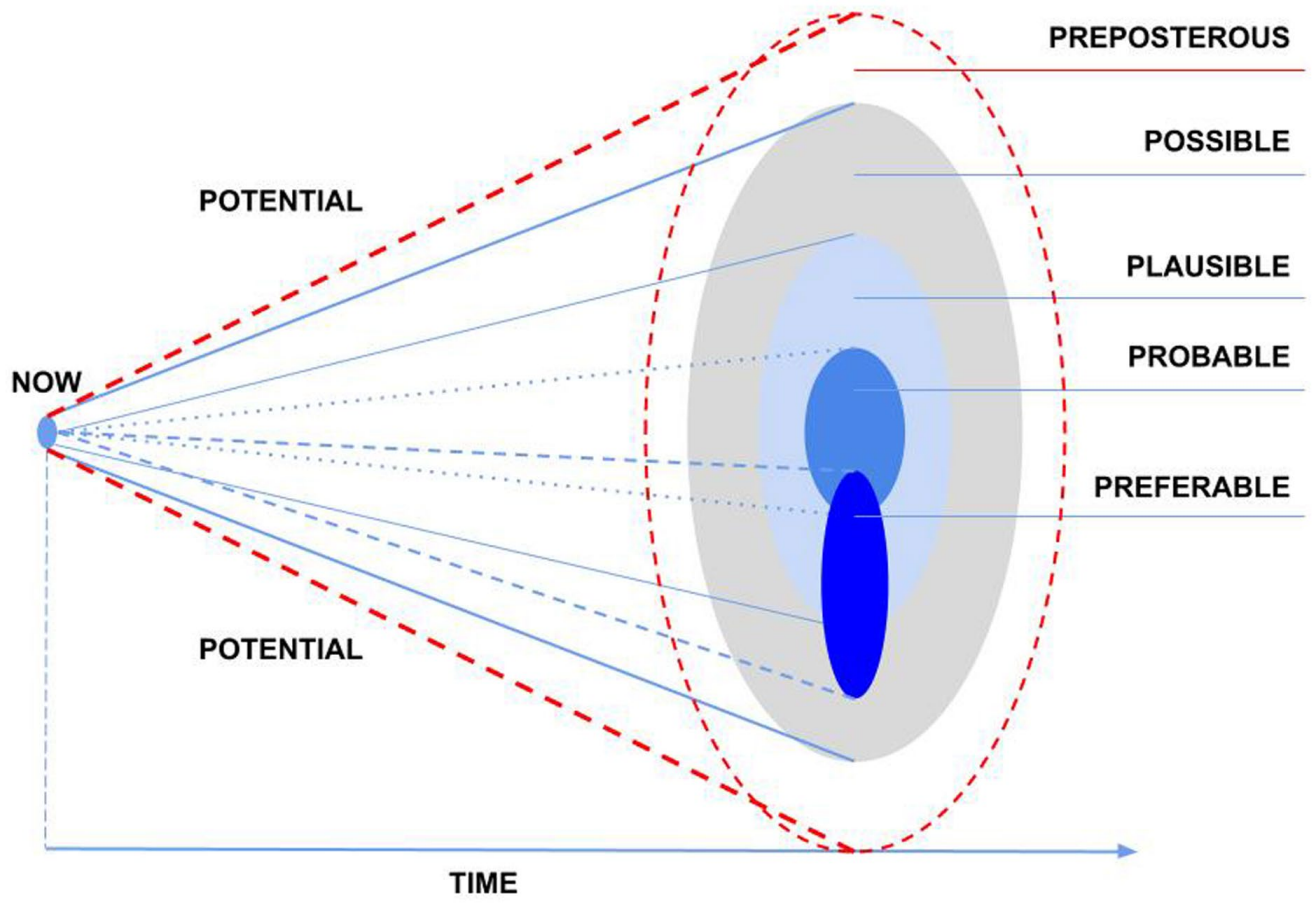
Futures Cone with preposterous Futures.

For many of us, because we work on impossible futures, the Futures Cone is irrelevant (Figure 2). All the preferred futures in the Futures Cone are possible, which is not our experience. The cone does not consider or include impossible futures. Even when futurists include an area or category called preposterous futures, it is only an extra thin layer outside the possible cone (Figure 3). More importantly, futurists only use a preposterous or impossible conical layer to indicate futures that are currently judged or assumed to be impossible but can move into the possible realm, like the moon landing (Voros, 2017). They do not mean impossible in the way Afropessimists and Afronihilists embody and experience impossibility. Afropessimists and Afronihilists literally believe that the very goal we desire, Black Liberation, is an actual impossibility—the shape-shifting, self-preserving, egotistical system of injustice will never allow it, though it changes its shape (Udoewa, 2024a).

**Figure 4:**
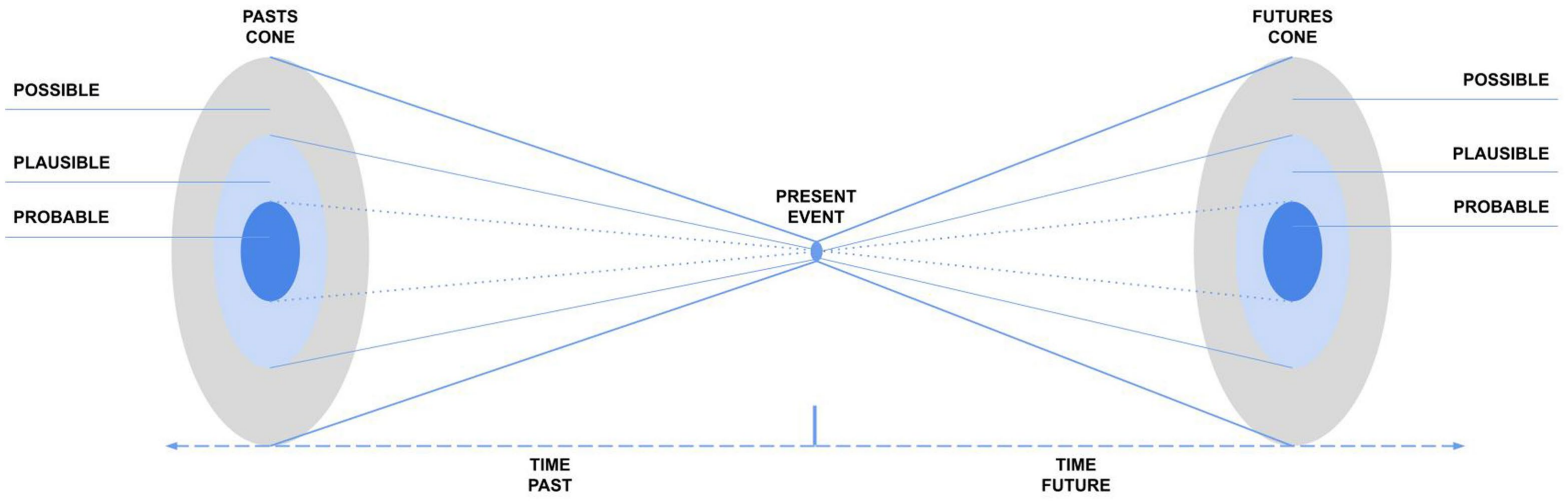
Alternative pasts and alternative Futures Cones.

There have been updates to the Futures Cone, such as recognizing that there are also alternative pasts (Christophilopoulos, 2021). Still, again, like alternative futures, alternative pasts do not go far enough in helping a community built on an ethic of hopelessness remain active toward a desired, yet impossible future (Figure 4). Alternative pasts are unhelpful because they are missing unacknowledged, disavowed, and prohibited pasts; they are missing impossible pasts that are linked to impossible futures. Impossible pasts are stories we and our ancestors have lived that the system dampens, buries, or rejects. For example, in the United States, there is growing white consciousness around racial tension that has always existed in the country, racial subordination upon which the country declared independence and built its wealth. Different states in the U.S. education system have been fighting to keep such Afrocentric narratives, stories, and worlds invisible and unheard to students, fighting over critical race theory, the purpose of the U.S. Civil War, and the U.S. Advanced Placement (AP) African-American History course. Governors of states like Florida banned the course, causing the U.S. College Board to remove topics like Black queer theory, radical Black feminism, critical race theory, and the Black Lives Matter movement and add Black conservatism to the course (Hartocollis and Fawcett, 2023). It is an impossible past that many of us social justice workers believe will never be allowed to be learned by all U.S. students in all U.S. schools.

Because we are working on futures that we experience as impossible, the Futures Cone is not useful. What, then, is a more useful approach to Futures for those of us around the world who desire impossible futures we will never see?

### Alignment, alternative presents, and alternative presence

One difficulty with the Futures Cone is its foundational, Western concept of time. The English word ‘time’ colloquially connotes a linear, cause-and-effect passage of events. Even when using the negative phrase ‘nonlinear time,’ writers and speakers are not saying what kind of time it is—cyclical, hyperbolic, elliptical, iterative, logarithmic, and so on. The phrase nonlinear time still reinforces the centrality or importance of time’s linearity. For many hyperlocal and Indigenous cultures, time is different from the Western understanding (Killsback, 2013; Mpofu, 2016; Adjaye, 2002). Similar to many people groups, in the world of my Ibibio peoples, we have a word for space and no one word for time. In order to translate the English word time, we have to hear the word in the context of a sentence or paragraph. Other people groups have a word for time and no word for space. Others have the same word for space and time. The way one experiences time depends on the world they inhabit.

Unfortunately, the English word ‘time’ miscommunicates the Ibibio experience. The better English word for our Ibibio experience with Western concepts of past, present, and future, is alignment. To use spatial terms, in the Ibibio world, our ancestors are not in a past place; our descendants are not in a future place. Our ancestors are here in this place with us. Our descendants are also here with us, as we are their ancestors. The question is not what did our ancestors experience in the past (hindsight), what is happening now (insight) and what could the future hold (foresight). Our question is alignment: Am I acting in alignment with the best, root intentions of my ancestors and the purest yearnings of my descendants? Are the communal values of my ancestors and the relational identities of my descendants acting as tailwinds, pushing me forward? Or are the wildest dreams of my ancestors and the flourishing freedoms of my descendants acting as headwinds, as I move in contradiction to them, working against them?

The question of alignment carries meaning because the past and the future, the ancestors and descendants are here with me in the present. Because my ancestors and descendants are here with me, I can be in relationship and dialogue with them. Just like a group of gathered people may try to move in alignment, so, too, do I and my ancestors and descendants try to move in alignment. This is the same understanding that enslaved African-Americans carried with them from Africa. Many African Indigenous people groups experience ancestors as being an active part of the present which made it easier for enslaved Africans to understand a resurrected Jesus who is active in the present today (Douglas, 2019; Stuckey, 2013).

If the ancestors and descendants are here, and the question before many Africans and African-Americans is alignment, then impossible pasts, impossible futures, and ultimately Black Liberation Futures are about the Western concept of the present. Our ancestors from impossible pasts are here, looking at us, talking to us Afro-descendant people.

*We have done and are doing our work. We have lived and are living faithfully. We have avoided defuturing you. We have done the work today so you, our preferred future, are more likely to happen. Now you must do your work. Be who you were meant to be in relation to the community who defines your being.*

Our descendants from impossible futures are also here, looking at us, talking to us.

*We have done and are doing our work. We have lived and are living faithfully to the impossible future. We have done the work today so you, our preferred past, are more likely to happen. Now you must do your work. Be who you were meant to be in relation to the community who defines your being.*

**Figure 5:**
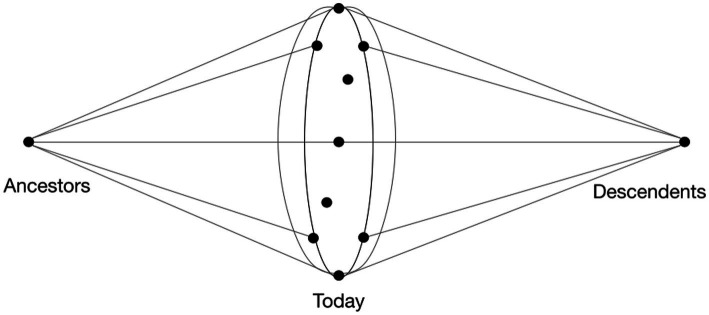
The cone of alternative and transcendent presents.

To use Western concepts, the past and the future have been taken care of by our ancestors and descendants. The only question and their only question, is who are we, the people of the present, going to be, today? Will we live consistently to their embodied impossible pasts and futures? In other words, Impossible Futures lead to alternative presents. We can choose to practice absencing, or avoiding, leaving, and going away from the responsibility of today and the power of now. We can also choose presencing, choosing to focus our awareness on now, be present, and dig into the story we are living today focusing on alignment. This is alternative presence, what Black Liberation Theology calls transcendent presents (Cone, 2022). We can live differently today choosing different presents (Figure 5).

Therefore, liberation is not a destination but a way of being—ontoliberationality. We see this, for example, in the life of the public mystic, freedom fighter, and womanist, Harriet Tubman (Page and Woodland, 2023; Taylor-Stinson, 2023). Born into U.S. American slavery, Harriet Tubman escaped, but did not consider her freedom as the end of liberation. She became the great Moses, the most well-known conductor of the Underground Railroad, leading at least 70 more people to freedom in the north, never losing a single passenger. Still not enough, she freed around 300 more through raids on plantations during the U.S. Civil War in the Combahee River uprising. Even when the Civil War ended and all U.S. American slaves were free, she spent the remainder of her freed life caring for the disabled, the elderly and the chronically ill. For Tubman, system healing and liberation were a way of life. She always practiced alternative presencing.

Alternative and transcendent presents and presence is exactly what community members learn from Black Liberation theology and eschatology. Redefining hope, Cone (2019, p. 114–115) says HOPE is not about patience, but impatience.^3^ Christians look to the future as a means of making themselves dissatisfied with the present (Cone, 2019, p. 142). The purpose of looking and communicating with a distant past or an unrealized, impossible future is to show the ungodliness of the present. This causes them to choose to be differently, choose an alternative present to live, choose to presence alternatively, transcendently. Desires in impossible futures must be turned into present realities.

Therefore, similar to scholar Derrick Bell’s racial realism, Black Liberation eschatology rejects the systemic trap of reformism and incrementalism because Black Liberation eschatology is about the present (Udoewa, 2024a; Cone, 2019; Bell, 1992b). Reflecting a deep Afropessimism, Black Liberation theology sees progress as irrelevant; African-Americans want Black Liberation now (Cone, 2019, p. 160, 165). It does not matter how many gains are won through civil rights because the face of the Black revolutionary will always exist as long as others define the bounds of Black being.

### The temporal forest

If Impossible Futures are not about progress but alternative presents and presencing, what is the relationship to the past? The past is very important for the African diaspora. Black Liberation is both deeply progressive and deeply conservative. Yet, we, African-Americans, for instance, are not trying to conserve any past in the United States. We are trying to conserve, preserve, and reserve uncovered and recovered African pasts before slavery. History making is relating, conversing, imagining, and designing that changes the way we relate to ourselves, things, narratives, and histories (Spinosa et al., 1999). When we uncover impossible pasts that the system does not want other people to know and learn—impossible pasts that are connected to impossible futures—we are making history. Therefore, similar to Critical Race Theory’s counter-storytelling, especially in Black Liberation work, there is no future-making without history-making (Delgado and Stefancic, 2023; Bell, 1987).

Often in White Futures there are various frameworks or high-level methodologies (Makelainen, 2023):

*Foresight → Insight → Action or Hindsight → Insight → Foresight*

White futurists uncover foresight through Futures data. That leads to insight about what to do today, and they take action. Or White futurists uncover past trends, extract insights from present events that then lead to foresight about the future. Though the past, present, and future are connected with blended transitions, Black Liberation Futures holds a more inextricably relational connection between the Western concepts of past, present, and future.

**Figure 6:**
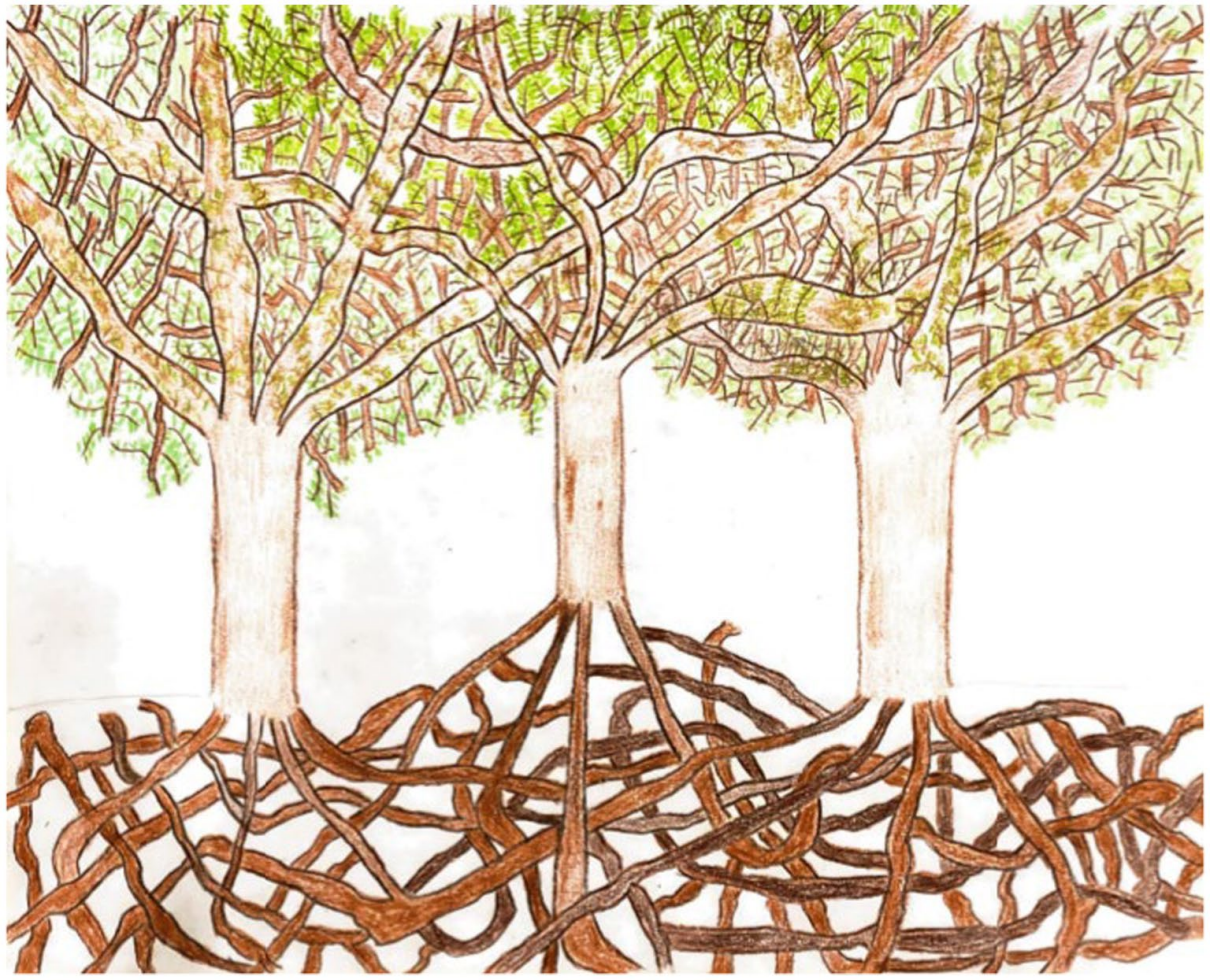
The temporal forest.

Compared to the Cone of Alternative Presents, a better visual metaphor for the African Indigenous experience of time is the Temporal Forest, which I describe lyrically to align with Ibibio ways of knowing and being (Figure 6). There are multiple trees: the Tree of Future Morrows, the Tree of Present Presence, and the Tree of Previous Pasts. All three trees are connected with intertwined and entangled branches. All three have subterranean roots that are entangled, grabbing each other for greater resilience during hurricanes or tornadoes. The roots of all three trees are interconnected through rhizomatic networks of mycelia that are sending messages between the trees, warning of impending danger and threats, and passing nutrients from one tree to any other tree that needs it. Most importantly, all three trees—the past, the present, and the future—are growing each year with a new ring of growth.

#### The tree of Future morrows

The future is always present. Through experiences like hypervigilance and paranoia, trauma shows us that the future can be present. All acts of yearning, imagining, and dreaming and planning are examples of the ways the future affects the present and guides our actions. Additionally our descendants are here with us creating continuity across generations.

The future is always past. The conservative progressivism of Black Liberation work exemplifies the past nature of the impossible futures. The longing for impossible futures incites internal, community excavation of our histories as we wonder if our ancestors faced similar struggles and what their life was like. As we uncover and discover past histories, the recovered past histories fuel new visions of the future exploding the transfinite possible future of the Futures Cone into the infinite impossible futures of the Temporal Forest.

#### The tree of present presence

The present is always future. Our present actions and work lay the memory and foundations upon which the future rests. Legacies of the present are left as gifts for the future. When the governments of South Carolina and Mississippi finally removed Confederate flags, legislators in the present were affecting the future education of students and experience of visitors and workers who will go to the capitol buildings (Wikipedia, 2025). The legislators changed the mythopoetic structures, narratives, and stories people will experience in the future. Those of us in the present are ancestors, speaking into the lives of those in the future, guiding them in dialogue.

The present is always past. The present is not just affected by the past, but affects the past. Like art, it is in the present that we visit and revisit the past and continue the work of shaking, sifting, reshaping and remaking, revising and reassessing, restorying and restoring the past. It is only the present understanding of the past that allowed the South Carolina and Mississippi legislators the ability to remove statues honoring Confederate soldiers from the public square.

Importantly, the Temporal Forest and Black Liberation Futures show that the present is much more than a conduit between the past and the future. As the present affects the future and past, more and more people—the hopeful, the hope-indifferent, the hope-unsure, and the hopeless—are invited into the inclusive ethic of hopelessness. As more and more people practice presencing, the community grows and explodes the finite number of presents the system allows into an infinite pluriverse of presents, growing the present foundation for history-making and future-making. And the Tree of Present Presence grows, ring by ring.

#### The tree of previous pasts

The past is always present. As silent ghosts, the past can haunt the present, for example, in trauma responses of numbness, insomnia, flashbacks and more. Various southern communities in the United States have had to learn and relearn what various Confederate symbols mean in the life of African Americans. Those past memories and transgenerational scars continue to inflict pain in the present. This is why many people called for the removal of Confederate statues from the public squares to museums, or the removal of Confederate soldier names from public buildings. Past institutions of slavery and the Jim and Jane Crow apartheid south live on in the U.S. in institutions like mass incarceration.

The past is always future. As stated earlier, in Black Liberation work, it is the image of precolonial convivialities that cause us to yearn for impossible futures (Hannah-Jones and Watson, 2021). Our future lies in reclaiming ancestral knowledge systems and learning from them. Despite the narrative that Indigenous and hyperlocal knowledge systems are stagnant, they continue to grow and learn. They can interact and dialogue with other knowledge systems.

The Tree of Previous Pasts does not grow only due to the passage of time, as more present becomes past. As we uncover, discover, and recover forgotten, dismissed, depressed, suppressed, and repressed pasts, impossible pasts, the tree grows to encompass more herstories and histories, ring by ring.

### Be the signal

In addition to time, signals are another area of difference between White Futures and Black Liberation Futures Design. In White Futures research, the Futures data comes from signals, trends, and drivers. Whether uncovered through ethnographic research, market research, or other sources, the signals and trends are used with various White Futures methods to create alternative future forecasts and scenarios. Practitioners and participants decide which forecasts or scenarios are preferred and why, then decide what strategies, tactics, and actions to employ in order to make the preferred futures more likely to happen and the unwanted futures less likely, or to make their organization more resilient no matter which future scenario occurs.

In Black Liberation Futures work, we—Black Liberation workers—already know the future we want: liberation. There may be slight differences in the details between different images of the future, but ultimately it is liberation with flourishing education, health, healthy food access, internet access, access to sustainable energy, labor, clean and sustainable environment, self-determination, sovereignty, and more. We do not need signals, Futures research, forecasts, and scenario planning to determine our preferred future. We already know.

As Black Liberation Futures Design is about system Futures, we use signals, instead, to anticipate how the systems of injustice will react to our liberation work and to be future-ready for the system trauma response (Udoewa, 2023). In 2005 and 2006, Edward Blum, the director of Project on Fair Representation, lobbied Congress and the White House to remove Section 5 of the Voting Rights Act (Gumbel, 2016; Hatcher-Mays, 2014). Section 5 of the act forced certain states with discriminatory histories to receive pre-clearance from the U.S. Department of Justice on new voting laws. Supported by conservative money, Blum had a history of taking cases all the way to the Supreme Court, winning victories in Texas, Florida, South Carolina, New York, Virginia, North Carolina, and Louisiana. His lobbying in 2005 could have been interpreted as a signal, forecasting a Supreme Court victory roughly a decade later arguing Section 5 was unconstitutional. If that had been done, southern communities of color, the youth, the LGBTQIA+ community, and the elderly could have strategically prepared for that scenario that actually occurred in 2013, eight years later.

In White Futures, signals help produce the forecasts and scenarios. However, sometimes there is no signal of impossible futures, another reason why White Futures approaches do not work well with impossibility. In Black Liberation Futures Design, we do not focus on looking for signals to forecast possible futures. We simply become the signal.

Becoming the signal is captured in the mantra ‘Be the change you wish to see in the world.’ We—impossible futurists—do not wait for a courageous person or group to be a signal or for other signal data to create a future or forecast we know we already want. We create and become the signal that others might map. The goal is to be the signal, which is an act of history and future making. Instead of waiting for the government to change laws or policy, we simply live a reality and world as if the impossible future we desire—liberation—has arrived and is here. By doing this, we liberate ourselves internally before any external freedom has occurred—another example of liberation as a way of being—ontoliberationality.

Being the signal is similar to the fractal politics of certain Black power and Black Feminist activists and scholars (Ritchie, 2023; Brown, 2017; Boggs and Kurashige, 2011). There is a similar focus on the small and transforming yourself. Being the signal in Black Liberation Futures goes further in a few ways. Activists Boggs, Ritchie, and brown focus on prefiguring a possible future that can come, while Black Liberation Futures focuses on impossible futures and being the signal for alignment. Also, the ethics of hopelessness is more inclusive and does not require a belief in the fractal emergence of a liberated future. Lastly, being the signal involves critical history-making with an archeological focus on impossible pasts.

Being the signal is exactly what Civil Rights Movement makers did in the U.S. in the 1960s. When African-Americans sat at lunch counters that were for whites only, waiting to be served, they were living as if the lunch counters were already integrated. When multi-racial groups rode buses together through the U.S. south in the Freedom Rides movement, they were living as if buses were already integrated. When Ruby Bridges and other African-American children were bussed to white schools, even while children and adults hurled objects and invectives at them, those African-American children were living as if schools were already integrated, even as the white school community reacted in hurtful trauma.

Ultimately, signal-being is not about sense-making. While White Design and Futures focuses on sense-making of data, Black Liberation Futures Design focuses on meaning-making. How do we live today that gives meaning to one another, in community, including our ancestors and our descendants? This carries a Black Liberation Theology sensibility in which solidarity with the oppressed and poor does not make colonial sense. However, God’s solidarity with Black people, the poor, and oppressed is profoundly meaningful for those groups.

These differences highlight how a Black Liberation Futures workshop based on the ethics of hopelessness in Impossible Futures differs from a White Futures workshop. First, we, Black Liberation futurists, spend time excavating impossible pasts; history-making is part of the work. Second, we do not focus on gathering Futures data research to forecast and imagine possible futures. We already know the impossible future we want. However, we do use signals to forecast people’s reaction to our work through embodied practices like role play or social presencing theatre (Udoewa and Dudani, 2024). Third, instead of sense-making, we focus on meaning-making, often non-sensically. In this way, we use a more values-sensitive design approach, explicitly spending initial relational time to name, decide, and define our values and then infusing them into every step, activity, practice, and method (Friedman and Hendry, 2019). Fourth, our work is not contained in or by workshops because it is transgenerational work across deep time. Many of us do this very work in the act of living and lack the privilege of stepping in and out of the work, at will. Fifth, because many of us come from non-Western ways of being and knowing, we use practices from other ways of being-knowing-doing—song, ceremony, dance, storytelling, role play, theater, learning circles, visual art, emotion, pilgrimages, music, intuition, dialogue, wailing circles, and more. Sixth, we often make decisions biocratically, not democratically (Udoewa, 2024b, 2024c). For example, instead of the majority deciding, we often make decisions for the minority embodying values of deep relationality and mutual care for those who are hurt. Lastly, as the fourth paper in this series will explicate, Black Liberation Futures work is inextricably relational (Escobar et al., 2024; Udoewa and Gress, 2023). We do not do this work with or as strangers, but in communities of interconnected lives, co-created and co-emerging with each other and our environments. Relationship-building is the work.

## Conclusion

This is the third paper in a paper series focused on learnings from Black Liberation work. In the first paper, readers explored a brief introduction to a healing-centered framework that defined system trauma (Udoewa, 2023). Part II shared learnings from specific system traps—system trauma responses—in Black Liberation (Udoewa, 2024a). This third paper explores how these consistent traps and entanglements in Black Liberation work affect the future orientation and emotional outlook of social justice workers, Black Liberation workers, and Afro-descendant people particularly.

In this paper, readers learned about an Impossible Futures practice, Black Liberation Futures Design, founded on an ethic of hopelessness. Africans, and thus humans, have always been futurists—from the intergenerational stone tool traditions of early hominids to the participatory Futures debates of African-Americans in 1862. There has been no distinct start of communities separate from Futures practice.

Next, readers explored Black Liberation Theology as an ethical lens through which readers viewed HCD and White Futures. Readers found that HCD and White Futures carry explicit ethics of hope, agency, and optimism in their values, mindsets, principles, and methods. Hope functions as a gateway requirement in order to practice HCD and White Futures, making them exclusive methodologies that epitomize a practice of becoming White—requiring community members and participants to become like the futurists and designers and their world. This violates the inclusive participatory eschatology of Black Liberation Theology. More importantly, it violates the Black Liberation Theology value of becoming Black. To be free, you must become Black—become linked in solidarity with people at the bottom of the social hierarchy, putting yourself in the same position, ready to face the same slings, arrows, and blows. In other words, futurists and designers should instead become Black and meet people where they are instead of creating exercises to train people to have more optimism, agency, and hope.

Through Afropessimism and Afronihilism, readers saw that there are people in the African diaspora who simply have no hope at all and find no meaning in this life in this current order in the social system in which they find themselves. The futures they desire are impossible; the system will never allow it. How can or do they engage in Futures when they are excluded from a White Futures practice that requires hope and agency? Readers explored how, in contrast to an ethic of hope, the ethic of hopelessness is an ethic that does not depend on hope at all. Inclusive of all relationships to hope—hope-unsure, hopeful, hope-indifferent, hopeless—the ethic of hopelessness is an ethic that moves people to action based on integrity, dignity, doing the right thing, right relationship with ancestors and descendants, and, ultimately, love.

Thus, with this ethic and a desire for impossible futures, the Futures Cone is irrelevant. I shared two different conceptions of time—the Cone of Alternative Presents and the Temporal Forest—that better connect to the experience of Afro-descendant people and Black Liberation work. Ultimately, alignment is a better English word for the African Indigenous experience of the Western concept of time. Lastly, Black Liberation Futures Design treats liberation as a way of being—ontoliberationality. The focus of such Futures work for impossible futures and impossible pasts is really alternative and transcendent presents and presence: how to be alternatively or transcendently in the present in alignment with ancestors and descendants. Instead of finding signals and sense-making, impossible futurists focus on meaning-making and being the signal.

This paper also carries direct methodological implications for sociological research on race and racism. First, it demonstrates the problem of scholastic bias in Futures-oriented race research: when sociologists and practitioners design participatory methods around race, temporality, and structural change, they routinely import epistemological assumptions about hope, agency, and linear progress. These assumptions are not neutral—they are racialized, and they reproduce the very categories and hierarchies they claim to study. Afropessimism and Afronihilism are not methodological barriers to be overcome through better training exercises; they are structurally produced orientations that reveal the limits of dominant methodological frameworks.

Second, this paper illustrates the importance of what the special topic calls epistemic reflexivity—awareness of how the researcher’s positionality, and the categories embedded in research practice, shape what knowledge about race becomes possible. My own social location as an Ibibio, African-American, invisibly disabled, immigrant-family person is not incidental to the arguments here; it is constitutive of them. The Ibibio concept of alignment, the Black Liberation theological lens, the standpoint advantages of those at the bottom of the social hierarchy—all of these shape the knowledge this paper describes. Sociological research on race requires the same reflexive awareness: that the theoretical and methodological tools researchers use are never simply tools; they are choices that determine whose temporal experience, whose relationship to the future, and whose ways of knowing become legible or invisible.

Third, Black Liberation Futures Design offers sociology a concrete methodological alternative for community-based research on race. Its emphasis on history-making alongside future-making (counter-storytelling as method), on meaning-making rather than sense-making, on embodied and relational ways of knowing, on biocratic rather than majoritarian or democratic decision-making, and on community relationship as the primary research infrastructure all speak to longstanding methodological conversations in qualitative sociology, critical race theory, and decolonial research methodology. Sociology can learn from these non-Western, community-rooted methodological traditions while maintaining its core analytical focus on social structures, processes, and meanings—not by importing them wholesale, but by allowing them to challenge the categories and techniques that have gone unexamined.

Other questions remain. What does it mean to take this system Futures orientation and move toward system health? Or is that even a goal in Black Liberation work, though it is a desire? From a systemic design framework, are there theories of change or specific levers used in the ontoliberationality that come out of Impossible Futures? What would it look like for sociology to institutionalize methodological reflexivity around hope and temporal assumptions in research design with racialized communities? These are questions to which researchers and practitioners can respond in future work.

## Footnotes

1 In this paper, Futures with an ‘F’ refers to a discipline, area of study, methodology, or practice of futuring. The word futures with an ‘f’ refers to images, scenarios, forecasts, and experiences of possible or impossible futures. In a few cases, the word ‘futures’ or ‘Futures’ refers to both, and in those cases the other ‘F’ or ‘f’ fits just as well.

2 In this paper, White or Whiteness with a ‘W’ refers to a way of being-knowing-doing. The word white refers to people of a specific, socially constructed, Eurocentric race, or a group of people of the same skin color.

3 In this paper, HOPE with all capital letters refers to a redefinition of hope different from a Eurocentric understanding that may be in a Western dictionary.
